# Prof. Peter Duesberg—Molecular Biologist *par excellence*. 2 December, 1936–13 January, 2026

**DOI:** 10.1038/s41375-026-02891-9

**Published:** 2026-02-25

**Authors:** Rüdiger Hehlmann

**Affiliations:** Medizinische Fakultät Mannheim, Universität Heidelberg, Mannheim, Germany

Prof. Peter Duesberg, molecular virologist and cell biologist at the University of California Berkeley was born in Münster, Germany. His father was Richard Duesberg, Professor of Medicine at Mainz University known for his shock-theory. Peter was educated in Germany and graduated in chemistry with Theodor Wieland at Goethe University in Frankfurt in 1963. After a brief stay at the Max-Planck-Institut (MPI) for Virology in Tübingen he moved to the University of California, Berkeley (UCB) in 1964.
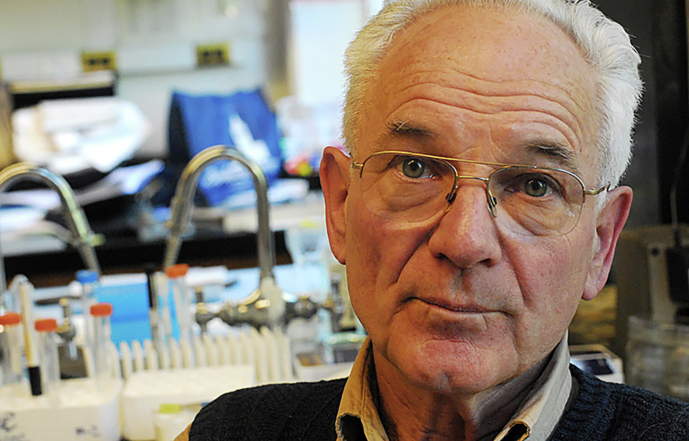


Duesberg’s detection of the segmentation of the influenza virus genome as a correlate of the high recombination and mutation rates of the influenza virus in 1968 [1] raised general attention. It was the prelude of his detection of viral oncogenes with Peter Vogt in 1970 [2].

These studies were widely recognized for their importance and in 1971, age 34, he was elected scientist of the year in California and in 1986, age 49, a member of the US-Academy of Sciences. From 1985 to 1992 he was recipient of an Outstanding Investigator’s Award from the US National Institutes of Health (NIH) followed by many other awards and honors.

He might have been awarded the Nobel Prize in Medicine or Physiology for detecting the first oncogene if - he had not been Peter Duesberg who remorselessly questioned positions which appeared inconclusive to him even if it concerned his own work. If it came down to scientific truth he was incorruptible. When candidates were considered for the Nobel Prize 1989 which in the end was given to his competitors H. Varmus and M. Bishoр from the University of California, San Francisco (UCSF) across the bay for the oncogene he had discovered, he just had challenged the relevance of proto-oncogenes for carcinogenesis in lectures and in a perspective article [3].

His quality to stick with an insight, which he had recognized for himself as true, has followed him through all his scientific life to the good—he was reliable, credible, consistent and calculable—but also to his disadvantage when the majority of his colleagues believed differently. Peter appeared conciliatory and easy going on the surface, but this did not apply when it came down to what he perceived as scientific truth. With his personality and as a chemist it may have been difficult for him to fully appreciate new biological avenues that contradicted classical rules.

I met Peter first in 1968 when he visited Peter Hans Hofschneider and his group in the MPI for Biochemistry in Munich. Peter, a charismatic speaker, reported on the segmentation of the influenza genome, and fascinated the group that we, Berthold Francke, Rudolf Jaenisch, I and others ultimately published reviews on human viruses filling a complete volume of the “Landarzt” for practicing physicians.

I still remember the fascinating lecture he gave on oncogenes and proto-oncogenes at the Munich Society for Morphology and Physiology in 1986. The lecture hall was overcrowded, everybody relevant in virus and cancer research was there. Peter did not need slides, using only chalk and black board. Although he spoke overtime, nobody left early.

Peter is listed in PubMed with 304 publications which is not much compared to what clinicians usually report. But allmost all publications are original papers with considerable scientific weight. There are very few reviews or re-hashes, and most manuscripts were written by Peter himself.

Unsatisfied by how the cause of AIDS was announced by the US Secretary of Health in 1984 and although he never worked experimentally on AIDS, Peter challenged the causative role of HIV, since the pathogenetic events leading from virus infection to AIDS were more than unclear at the time. To provide unbiased and scientifically correct information on AIDS, Peter and others including Bob Gallo, joined the editorial board of AIDS-Forschung, a journal which I edited from 1986 to 1996 as a forum for AIDS.

The causative role of HIV was ultimately established on epidemiological grounds which, however, was not to Peter’s taste as a chemist and experimental scientist, and he remained unsatisfied. He published his views in articles and a book ‚Inventing the AIDS virus‘ (1996). Thus, unfortunately, the battle went on.

Stubbornness and consistency are two closely related virtues. There are examples in history: When Socrates was charged from the senate because of ‘incessant, irksome, and irritating questioning of all aspects of existence as a deliberate perversity’, he answered that he shall not cease to practice philosophy as long he is able to breathe.

There is, indeed, strong agreement in the scientific community that scientific debate is the centerpiece of scientific progress. Peter’s friends admired his courage and steadfastness in scientific and public debate regardless whether they shared his views or not.

In 1996 Peter called me in Mannheim saying that he was unsatisfied with our understanding of carcinogenesis and suggested to look at chromosomes and aneuploidy as proposed by Boveri 100 years ago. Based on this, my faculty invited Peter to serve as a Guest Professor at the Mannheim Medical Faculty of Heidelberg University where he stayed on and off for almost 10 years. He found that genetic instability of cancer cells is proportional to their degree of aneuploidy [4], that aneuploidy correlates 100% with chemical transformation, and that the high mutation rates of cancer cells to drug and multidrug resistance are explained by chromosome reassortments that are catalyzed by aneuploidy [5]. Based on these findings aneuploidy moved from specialty research to mainstream. Currently no leukemia is diagnosed anymore without karyotyping.

Peter was an enthusiastic and inspiring experimentator. He loved to do his experiments by himself. He had two golden hands and a clear straight forward mind when planning his experiments. I never observed that an experiment failed on technical grounds.

Peter accomplished much in his life, more than many of us could dream of. The identification of oncogenes like *src*, *myc* or *myb* was a milestone, probably his greatest achievement. With aneuploidy in cancer and its similarity to speciation, he addressed another important principle in biology, and he saw a connection between oncogenes and aneuploidy: Chronic myeloid leukemia offers an example for the cooperation between an oncogene (*BCR::ABL1*) that induces genetic instability and aneuploidy indicating progression to blast phase.

Peter retired in 2022 at age 85. He is survived by 4 children from 2 marriages: 3 daughters (Nicola, Susanne, Sibyl) and one son (Max), 2 grandchildren, and his wife Siggi who faithfully took care of him, particularly in the end.

He is remembered by his friends for his scientific brilliance, his youthfulness and openess for discussion, his persistence, his personal courage and his clear mind.

Rüdiger Hehlmann, Weinheim, Germany
